# Soybean (*Glycine max* L. Merrill) responds to phosphorus application and *rhizobium* inoculation on Acrisols of the semi-deciduous forest agro-ecological zone of Ghana

**DOI:** 10.7717/peerj.12671

**Published:** 2022-03-02

**Authors:** Samuel Adjei-Nsiah, David Martei, Adam Yakubu, Jacob Ulzen

**Affiliations:** 1 Forest and Horticultural Crops Research Centre, College of Basic and Applied Sciences, University of Ghana, Accra, Ghana; 2 International Institute of Tropical Agricultural (IITA), Ibadan, Nigeria

**Keywords:** Grain yield, Rhizobia, Value cost ratio, P-fertilizer, Residual effect

## Abstract

Soybean cultivation in Ghana is limited mainly to the Guinea savanna and the forest/savanna transitional agro-ecological zones. Although soybean can be cultivated in the semi-deciduous forest zone, low soil pH and limited nodulation limit its productivity in this zone. In this study, a randomized complete block design, with four replications, was used to test if rhizobia inoculation and/or p-fertilizer could improve yield of soybean in the semi-deciduous forest zone. The residual effects of the treatments were tested on maize and soybean sequentially during the 2018 and 2019 cropping seasons. The inoculation study was repeated in 2020. Phosphorus and inoculation significantly (*p* = 0.0009) increased soybean grain yield in the 2018 cropping season by 88% and 108%, respectively over the control. Co-application of P and inoculant increased grain yield 3-fold over the control. Maize grain yield ranged from 4.3 t ha^−1^ in the control plots to 5.2 t ha^−1^ in treated plots but did not differ significantly among treatments. In 2020, the combined application of P and inoculant produced a significantly (0.002) higher yield than any of the other treatments. This demonstrates that soybean can be grown economically in the semi-deciduous forest agro-ecological zone of Ghana. Co-application of P and inoculant appeared cost-effective, in terms of return on investment.

## Introduction

Soybean (*Glycine max* (L.) Merrill) is becoming an important cash crop in Ghana. The crop has a high nutritional value in terms of protein (32–42%) and oil (18–20%) (Liu, 1997). Soybean can also improve soil fertility owing to its ability to fix atmospheric N in symbiotic association with rhizobia (Sanginga, 2003; Osunde et al., 2004). Soybean reduces the need for mineral N fertilizer to the subsequent cereal crop grown in rotation (Carsky et al., 1997; Gentry et al., 2001; Sanginga, 2003; Osunde et al., 2004). Integration of soybean in the predominantly cereal-based farming systems in sub-Saharan Africa offers a pathway for sustainable intensification.

Soybean cultivation in Ghana is limited to the savanna and the forest-savanna transitional agro-ecological zones, where it has received extensive research attention in terms of legume-rhizobia technology. The crop is rarely grown in the semi-deciduous forest agro-ecological zone of Ghana due to limited nodulation and poor yields. Recently, soybean cultivation has extended into the forest zone of Ghana where double cropping is feasible due to two growing seasons; however, absence of effective rhizobia in the soils of the semi-deciduous forest appears to result in poor nodulation and low yield. Introduction of legumes into tropical areas, therefore, requires the application of rhizobium inoculant (Date, 2000) and/or p-fertilizer. Phosphorus often controls nitrogen fixation by legumes (Pérez-Fernández, Míguez-Montero & Valentine, 2019) and this macronutrient is sparingly available in soils in Ghana (Masso et al., 2016; Buri et al., 2010).

Inoculation of seeds with rhizobium strains enhances nitrogen fixation in grain legumes (Ahiabor et al., 2014; Asei, Ewusi-Mensah & Abaidoo, 2015; Masso et al., 2016; Ronner et al., 2016; van Heerwaarden et al., 2018; Adjei-Nsiah et al., 2018; Ulzen et al., 2018; Osei et al., 2020). Yield increases range between 50% and 100% when phosphorus fertilizer is applied together with rhizobia inoculation (Ronner et al., 2016; Adjei-Nsiah et al., 2018). Soil type, pH, organic C, and soil N influence nodulation and the overall results of rhizobia and/or p-fertilizer studies on soybean. For instance, soils in the semi-deciduous forest zone have low pH. Thus, it is important to determine how soybean will respond to rhizobia and/or p-fertilizer in a semi-deciduous forest agro-ecological zone.

In this study, we evaluated the effects of rhizobia inoculation and phosphorus application on nodulation and grain yield of soybean in the semi-deciduous forest agro-ecological zone of Ghana. Subsequent effects on maize and soybean grown afterward and the economic feasibility of growing soybean in the forest zones of Ghana was assessed. Inoculation with rhizobia and co-application of rhizobia and p-fertilizer resulted in significantly higher yield increases in 2018 and 2020. Rhizobia inoculant and/or p-fertilzer was cost effective with higher value cost ratios (VCRs) above the threshold of 2.

## Material and method

### Study site

The trial was conducted at the Forest and Horticultural Crops Research Centre, Kade (6°09′ N, 0°55′ W) in the Denkyembour district of the Eastern Region of Ghana. The centre is in the semi-deciduous forest agro-ecological zone of Ghana and is 114 m above sea level. The study site is characterized by a bimodal rainfall pattern with a 30-year average of 1,433 mm. The major growing season is from March to July and the minor growing season is from September to November followed by a dry season from December to February. Cumulative rainfall amounts during the study period were 711 mm in 2018, 935.5 mm in 2019 and 281 mm in 2020 with 35, 92 and 24 rainy days, respectively. Soils at the experimental site were developed from precambium phyllitic rocks (Ahn, 1961) and are deep, well-drained and classified as Gleyic Acrisols in the FAO-UNESCO Revised Legend (FAO, 1998). Soil sampling was carried out at the start of the experiment at 0–20 cm depth using an auger. Soil pH was determined according to the electrometric method in distilled water at a ratio of 1:1 (Table 1). Total nitrogen, organic carbon, and available P (Table 1) were analyzed using standard methods (Bremner & Mulvaney, 1982). Rhizobia count was estimated through the most probable number technique (Somasegaran & Hoben, 2012) as previously described by Ulzen et al. (2016). Soybean seeds were used as the trap host. Pre-germinated soybean seeds were inserted into a growth pouch filled with Dilworth N-free nutrient solution. A six step, five-fold serial dilution was prepared from the soil. One millilitre of the solution was used to inoculate the seeds. The pipette tips were changed for every step to prevent cross-contamination. The nodulation pattern was assessed after 28 days for the presence or absence of nodules. Rhizobia population was then estimated by MPNES software (Woomer, Bennett & Yost, 1990).

**Table 1:** Physico-chemical properties of the soil at the study site.

Properties	Mean values
Sand (%)	78.58
Silt (%)	7.84
Clay (%)	13.58
Textural class	Sandy loam
pH (Water) (1:1)	6.09
Total N (%)	0.19
Organic Carbon (%)	1.32
Available Phosphorus (mg kg^−1^)	10.59
Exchangeable Ca (cmol _(+)_ kg^−1^)	2.4
Exchangeable Mg (cmol _(+)_ kg^−1^)	0.68
Exchangeable K (cmol _(+)_ kg^−1^)	0.49
Rhizobia cell g^−1^ soil	11.4

### Experimental layout

The experiment was conducted in a randomized complete block design with four replications. The experimental field had been fallowed for 3 years and was cropped to maize for one season before planting soybean. The field was slashed with a cutlass and sprayed with glyphosate (36% active ingredients) at the rate of 2.5 L per hectare. Plot size was 6 m × 5 m with planting spacing of 50 cm × 10 cm. There was 1 m spacing between plots, while 1 m space was left between replicates. The soybean variety used was TGx 1448-2E locally known as “Jenguma” which means “wait for me” due to its non-shattering nature. “Jenguma” has a potential yield of 2.5 t ha^−1^, matures between 110 and 115 days and is resistant to striga (Dugje et al., 2009). The treatments evaluated were: control; Phosphorus (P) only; Inoculation (I) only; and Phosphorus and Inoculation combined (P + I). Phosphorus was applied as Triple super phosphate (46% P_2_O_5_) at the rate of 20 kg P ha^−1^. Phosphorus fertilizer was applied at planting in furrows 10 cm away from the planting line. The rhizobium inoculant used was Nodumax® and contained 1 × 10^3^ cells g^−1^ of *Bradyrhizobium japonicum* strain USDA 110 manufactured by the International Institute of Tropical Agriculture (IITA), Ibadan, Nigeria. In 2018, soybean was planted on 3rd September 2018. The two-step method of inoculant application previously described by Adjei-Nsiah et al. (2019) was used. Specifically, soybean seeds were moistened with Gum Arabica solution in a basin and the inoculant was added at the rate of 10 g per kg of seeds. The mixture was stirred thoroughly and uniformly with a wooden spatula until even coating was attained. Seeds were then spread on a sack under a shade and allowed to air dry for 30 min to enable the inoculant to stick the seeds. Treated seeds were sown at two seeds per stand early in the morning to avoid exposure to direct sun rays that might affect the quality of the inoculant. Uninoculated seeds were sown before the inoculated ones to avoid contamination. Weeds were controlled with herbicide-Vezir 240 SL at 50 ml/15 l water.

### Nodulation, shoot biomass and chlorophyll assessment of soybean crop in 2018 and 2019 minor cropping seasons

Nodulation and shoot biomass assessment follows similar pattern previously described by Adjei-Nsiah et al. (2019), Ulzen et al. (2016). Specifically, at 50% flowering (R_2_ growth stage), 10 plants were randomly uprooted and cut at the soil level from each plot; the root zone was gently washed on a 2 mm mesh sieve under tap water. Nodules were counted, and together with the shoot, oven-dried at 65 °C for 48 h to determine their dry weights on standard electronic scale. Chlorophyll concentration of the soybean leaves was determined using a portable chlorophyll meter (Model CCM-200 Plus; Apogee, Santa Monica, CA, USA).

### Yield assessment of soybean crop in the 2018 and 2019 minor cropping season

Physiological maturity occurs when 95% of the plants change from green to golden yellow (Tukamuhabwa et al., 2002) and about 75% of seeds are hardened (Masumba, 1984). Plants were harvested at this stage from an area of 16 m^2^ (8 rows of 4 m length) from 30 m^2^ plot leaving 100 cm from the outer ends of the plot. After harvesting, 10 plants were randomly selected for podding capacity. Pods were detached from the soybean plant and counted. The average number of pods per plot was determined. After threshing, grain and haulm yields were determined and the haulm was returned to the respective plots.

### Planting of maize test crop

On May 5, 2019, maize variety Pioneer (Pioneer is high yielding; up to 12 kg ha^−1^, has good grain colour, big ear and shelling and matures within 105–115 days) was planted on all the plots previously cultivated to soybean at a spacing of 50 cm by 30 cm at 1 seed per stand to evaluate the residual effect of the soybean on the subsequent maize crop. Prior to planting the maize, the plots were slashed with cutlass and sprayed with glyphosate 2 weeks later. Weeds were manually controlled in the maize plots at 4 and 8 weeks after sowing.

### Maize yield assessment

At maturity, maize ear and stover were harvested from the 10 middle rows leaving 60 cm border at both ends. The cobs were weighed and a sub-sample of 10 cobs per plot were taken, weighed and oven dried at 70 °C for 48 h for dry matter determination. The grains were extracted and weighed again to determine the dry matter (DM). The stover was however, weighed in the fresh state and sub-sample taken to determine the DM.

### Planting of the second soybean crop

On September 1, 2019, 3 weeks after harvesting the maize, the field was sprayed with glyphosate and soybean variety TGx 1448-2E locally known as “Jenguma” was sown on all the plots at a spacing of 50 by 10 cm at 2 seeds per stand to again assess the residual effect of the previously cropped soybean on the second soybean crop. Nodulation and grain yield were assessed as previously described under the 2018 minor cropping season experiment.

### Soybean planting for 2020 minor cropping season

The 2020 experiment was carried out on September 30, 2020, in the minor growing season on a different plot within the same location on a different experimental field. Plot size, plant spacing, variety planted, and cultural practices were the same as those of 2018 and 2019 plantings. The treatments were control; Phosphorus (P) only; Inoculation (I) only; and Phosphorus and Inoculation combined (P + I). Similar nodulation and yield data were collected as described under the 2018 experiments.

### Value cost ratio analysis

Profitability of applying rhizobia inoculant and/or p-fertilizer for the 2018 soybean planting was evaluated through the value cost ratio (VCR) equation (Roy et al., 2006).

$${\rm{VCR = }}{{{\rm{Value}}\;{\rm{of}}\;{\rm{extra}}\;{\rm{crop}}\;{\rm{produced}}\;{\rm{due}}\;{\rm{to}}\;{\rm{treatment}}\;(\$ \;{\rm{h}}{{\rm{a}}^{{\rm{ - 1}}}})} \over {{\rm{Cost}}\;{\rm{of}}\;{\rm{treatment}}\;(\$ \;{\rm{h}}{{\rm{a}}^{{\rm{ - 1}}}})}}$$

Rhizobia inoculant and TSP fertilizer cost 16 and 86 US$ ha^−1^ on the market, respectively. Soybean was sold at 0.44 USD$ kg^−1^ from the open market (Ulzen et al., 2020). As previously described by Roy et al. (2006), a VCR value ≥ 2 is considered profitable, VCR ≤ 1 is not profitable. The dollar to cedi exchange rate as at the time of this study in 2018 was USD$ 1 to GH¢4.5 and in 2019 was USD$ 1 to GH¢5.0.

### Statistical analysis

Shapiro–Wilk’s test at 5% probability was used to check the normality of soybean and maize data. Analysis of variance (ANOVA) was applied to the soybean and maize data using SISVAR version 5.6 (Ferreira, 2008). Treatment means were separated with Scott Knott test at 5% probability. The yield data from 2018 and 2020 was pooled together to examine the effect of year on the treatments. Robust regression was fitted to soybean grain yield in 2018 and chlorophyll content, respectively.

## Results

**Figure 1:**
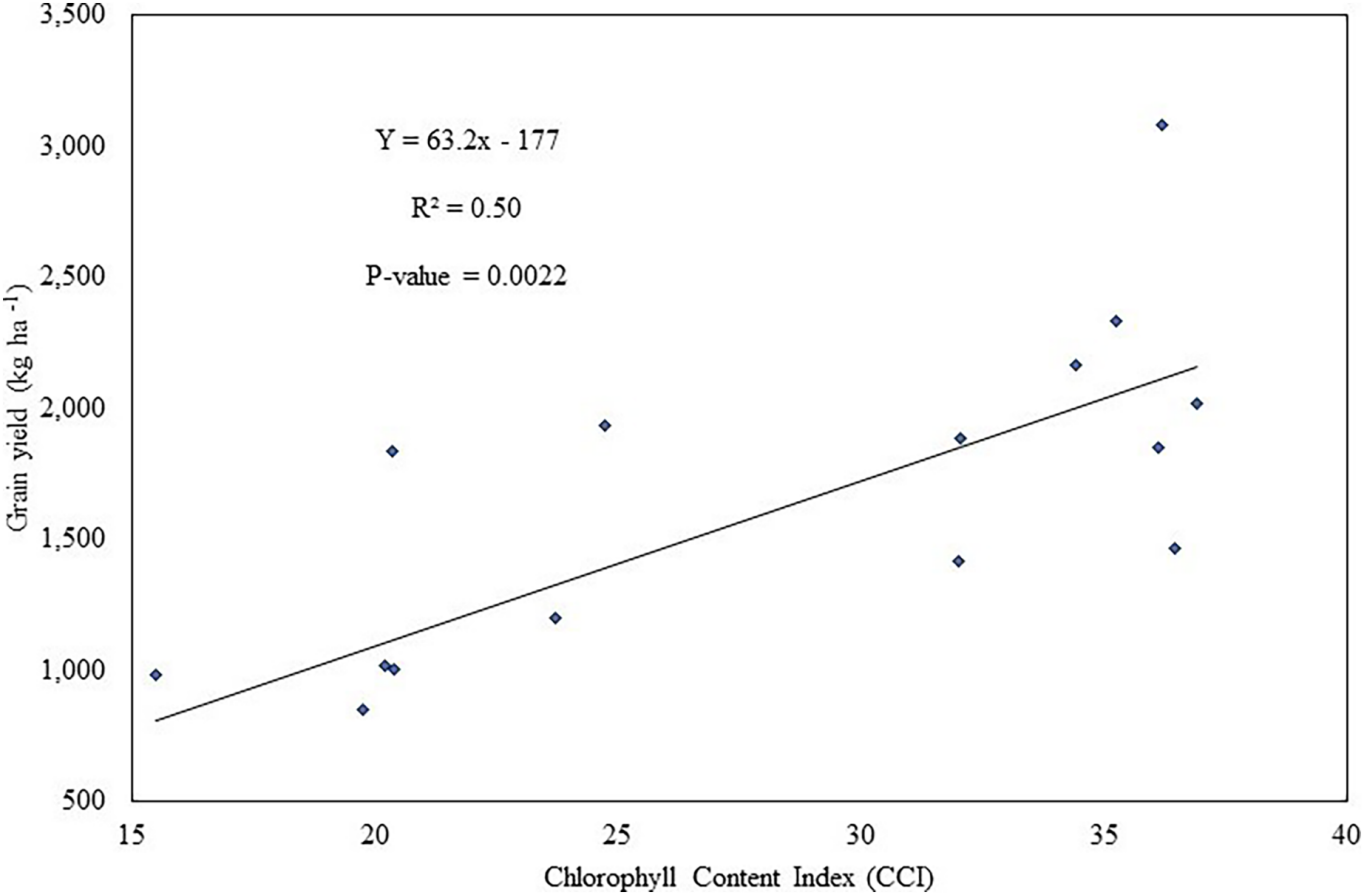
Relationship between soybean grain yield and chlorophyll content index in 2018 cropping season.

Soybean yield in 2018 increased markedly with the application of inoculant and/or p-fertilizer compared to the control (Table 2). Phosphorus fertilizer and inoculation effect resulted in 88% and 108% increase in grain yield over the control. Co-application of p-fertilizer and inoculant increased grain yield of soybean by 3-fold over the control. The co-application of inoculant and p-fertilizer significantly (*P* = 0.0009) increased soybean grain yield by 45% and 60%, respectively over plants that received inoculant and p-fertilizer alone in the 2018 cropping season (Table 2). No significant difference was observed between plots that received inoculant and p-fertilizer alone. A robust regression analysis between soybean grain yield in 2018 and chlorophyll content index showed that 50% of the variation in 2018 soybean grain yield was significantly (*P* = 0.0022) accounted for by the chlorophyll content in the plants (Fig. 1).

**Table 2:** Soybean response to inoculation and/or p-fertilization in 2018 minor season.

Treatment	Grain yield (kg ha^−1^)	Stover yield (kg ha^−1^)	Pod number plant^−1^	Nodule number plant^−1^	Nodule dry weight mg plant^−1^	Chlorophyll content index (CCI)
Control	796 ± 152*^,^^†^^,c^	2,500 ± 617^b^	18.0 ± 0.91^c^	0.0 ± 0.0^c^	0.0 ± 0.0^c^	19.48 ± 1.18^b^
TSP (P)	1,496 ± 228^b^	4,863 ± 948^a^	43.0 ± 6.78^b^	8.0 ± 8.25^c^	1.14e^−13^ ± 0.0^c^	22.27 ± 1.84^b^
Inoculant (I)	1,654 ± 123^b^	3,317 ± 406^b^	42.0 ± 9.34^b^	147 ± 26^b^	568 ± 121^b^	34.49 ± 1.27^a^
Inoculant plus TSP (I+P)	2,400 ± 237^a^	5,258 ± 307^a^	64.0 ± 4.02^a^	270 ± 55^a^	1,850 ± 336^a^	35.46 ± 0.85^a^
*P*-value	0.0009	0.017	0.0039	0.0011	0.0001	0.0001

**Notes:**

† Means within column, with the same letters, are not different at 5% probability level (Scott Knott Test).

* Standard error of the mean,

Stover yield follows a similar pattern as the grain yield in 2018. The combined application of inoculant and p-fertilizer and p-fertilizer alone elicited significant (*P* = 0.017) higher Stover yield of 110% and 95%, respectively over the control (Table 2). Application of inoculant alone, without p-fertilizer, did not increase stover yield (Table 2).

Application of inoculant and/or p-fertilizer significantly increased soybean pod number. The combined application of inoculant and p-fertilizer produced as many as four-folds of the pod number produced by the control plots and about 1.5 folds of the pods produced by inoculant and p-fertilizer alone. This increase in pod number produced by inoculant (133%) and p-fertilizer (139%) was significant (Table 2).

Inoculation alone, and combined application of inoculant and p-fertilizer, increased nodulation (Table 2). Control plots did not produce any nodules. Combined application of inoculant and p-fertilizer increased nodule number 34-fold over that of the p-fertilizer alone. Similarly, application of inoculant alone increased nodule number 18-fold over that of p-fertilizer alone. Nodule dry weight followed a similar pattern. Plots that received inoculant alone and combined application of inoculant and p-fertilizer showed significantly (*P* = 0.0001) higher nodule dry weight over plots that received p-fertilizer alone (Table 2).

Combined application of inoculant and p-fertilizer increased content chlorophyll in plants significantly (*P* = 0.0001) relative to plants that received p-fertilizer alone and control (Table 2). Plants that received a combined application of inoculant and p-fertilizer and inoculant alone, respectively produced 82% and 77% more chlorophyll than that of the control. Similarly, inoculated plants and plants that received co-application of inoculant and p-fertilizer significantly (*P* = 0.0001) increased chlorophyll content index by 55% and 59%, respectively over plants that received p-fertilizer alone (Table 2).

Inoculation and ferilization had little residual effects in terms of grain yield the subsequent year. In 2019, the number of maize cobs harvested did not differ significantly (*P* = 0.06) among treatments (Table 3). Plots that had previously received p-fertilizer produced the highest maize grain yield but this increase was not significant (*P* = 0.48). Rhizobia inoculation and/or p-fertilization did not show any significant residual effects on soybean nodule number and grain yield (Table 3). Plots that had not received inoculant in 2018, formed nodules in 2019 (unlike in 2018).

**Table 3:** The residual effect of inoculation and p-fertilizer on maize growth and yield 2019 minor season.

Treatment	Maize Grain yield (kg ha^−1^)	Maize cob number per plot	Soybean Grain yield (kg ha^−1^)	Soybean Nodule number plant^−1^
Control	4,353 ± 241*^,^^†^^,a^	49 ± 2^a^	667 ± 117^a^	25 ± 13^a^
TSP (P)	5,232 ± 324^a^	55 ± 4^a^	817 ± 116^a^	27 ± 8^a^
Inoculant (I)	4,962 ± 353^a^	56 ± 4^a^	975 ± 108^a^	22 ± 8^a^
Inoculant plus TSP (I+P)	5,204 ± 608^a^	62 ± 2^a^	1,075 ± 132^a^	33 ± 11^a^
*P*-value	0.48	0.06	0.21	0.87

**Notes:**

† Means within a column, with the same letters, are not different at 5% probability level (Scott Knott Test).

* Standard error of the mean.

**Figure 2:**
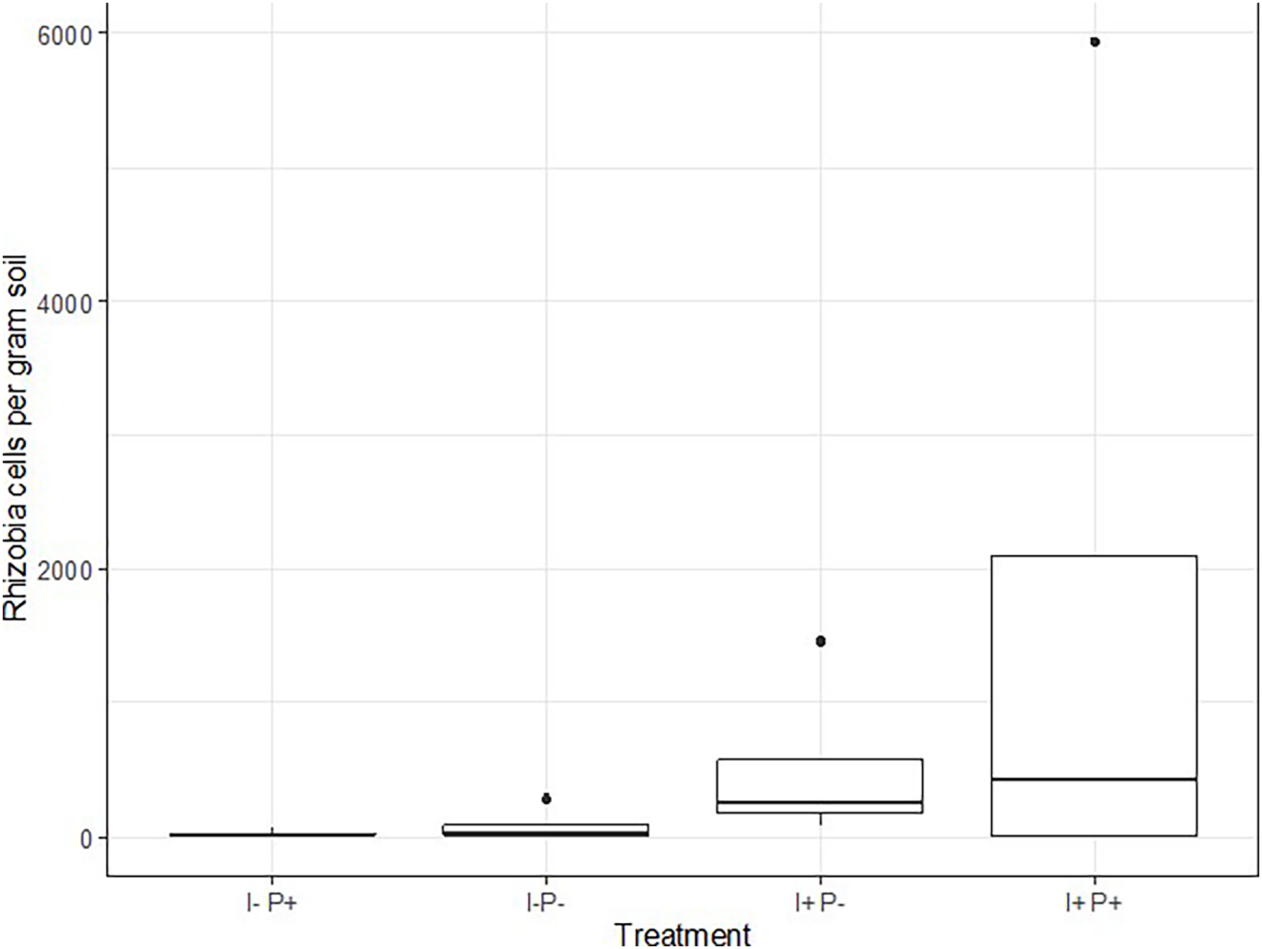
Rhizobia count in 2019 cropping season.

Rhizobia cell count suggested a residual effect of inoculation the previous year. The median rhizobia cell count in samples collected in 2019 from plots that received inoculant alone in 2018 was 251 cell g^−1^ soil and the median count in samples collected in 2019 from plots that received inoculant and p-fertilizer in 2018 was 830 cells g^−1^ soil in plots (Fig. 2). In contrast, the median rhizobia cell counts in plots that had previously received the p-fertilizer alone was 22 cells g^−1^ soil and 21 cells g^−1^ soil for control plots.

The benefit observed in 2018 of combined inoculation and p-fertilization on soybean yield was confirmed in 2020; however, grain yield of soybean in 2020 was lower than that of 2018 and 2019 (Table 4). Combined application of inoculant and P increased grain yield by 134% (Table 4). Stover yield followed the same pattern as the grain yield. Only the combined application of the inoculant and P-fertilizer significantly increased stover yield (*P* = 0.033). Control plots and plots that received p-fertilizer, only did not nodulate. Only treatments that received inoculant only and combined inoculant and p-fertilizer nodulated. Combined application of inoculant and p-fertilizer increased nodule number and nodule dry weight by 144% and 171%, respectively, over inoculant alone.

**Table 4:** Soybean response to inoculation and/or p-fertilization in 2020 minor season.

Treatment	Grain yield (kg ha^−1^)	Stover yield (kg ha^−1^)	Nodule number plant^−1^	Nodule dry weight mg plant^−1^
Control	417 ± 81*^,^^†^^,b^	617 ± 140^b^	0.0 ± 0^c^	0.0 ± 0^c^
TSP (P)	492 ± 99^b^	750 ± 142^b^	0.0 ± 0^c^	0.0 ± 0^c^
Inoculant (I)	547 ± 91^b^	777 ± 201^b^	105 ± 18^b^	850 ± 194^b^
Inoculant plus TSP (I+P)	1,280 ± 118^a^	1,406 ± 218^a^	256 ± 17^a^	2,300 ± 402^a^
*P*-value	0.002	0.033	0.0003	<0.0001

**Notes:**

† Means within a column, with the same letters, are not different at 5% probability level (Scott Knott Test).

* Standard error of the mean.

Value cost ratio analysis showed that all the treatments were economically viable and profitable on soybean in 2018 (Table 5). Although, in 2020, the effect of rhizobia inoculant resulted in the highest VCR, the treatment that generated the highest revenue was the combined application of inoculant and p-fertilizer. Co-application of inoculant and p-fertilizer had the highest VCR of 4.5, followed by inoculant alone with a VCR of 3.7. P-fertilizer alone was not profitable in 2020, as it had a VCR of 0.5. Revenues generated from the residual effects of the treatment on soybean in 2019 followed a similar pattern as the revenue generated in 2018 (Table 5). The combined application of rhizobia inoculant and p-fertilizer had the highest revenue whiles the p-fertilizer alone recorded the least revenue. However, revenue from the residual effects of the treatment on maize followed a different pattern from that of soybean. Revenue generated from maize grown on plots that previously received p-fertilizer was the highest (Table 5).

**Table 5:** Value cost ratio and revenues generated from the residual effect of the treatments.

Value cost ratio 2018 cropping season				
Treatment	Yield gain (kg ha^−1^)	Revenue (USD$ kg^−1^)	Cost (USD$ kg^−1^)	VCR
Inoculant (I)	858	381.33	15.56	25.0
Inoculant plus TSP (I+P)	1,604	712.89	85.78	8.0
TSP (P)	700	311.11	70.22	4.0
**Value cost ratio 2020 cropping season**				
Inoculant (I)	547.30	57.72	15.56	3.7
Inoculant plus TSP (I+P)	1,280.15	383.47	85.78	4.5
TSP (P)	491.65	32.98	70.22	0.5
**Net revenue from residual effect on soybean in 2019 minor cropping season**				
Inoculant (I)	308	123		
Inoculant plus TSP (I+P)	408	163		
TSP (P)	150	60		
**Net revenue from residual effect on maize in 2019 major cropping season**				
Inoculant (I)	609	244		
Inoculant plus TSP (I+P)	851	340		
TSP (P)	879	352		

## Discussion

This study confirmed that rhizobia inoculation can significantly increase grain yield of soybean. Response to inoculation depends on several factors; prominent among these factors include indigenous rhizobia population and initial soil nitrogen (Thies, Singleton & Bohlool, 1991). The indigenous rhizobia population of the study location was 11 rhizobia cells g^−1^ soil, which is less than the threshold of 50 cells g^−1^ soil (Slattery, Pearce & Slattery, 2004) and 100 cells g^−1^ soil (Thies, Singleton & Bohlool, 1991) to prevent significant response to rhizobia inoculation. The response of soybean to rhizobia inoculation indicates that the initial amount of nitrogen at the study location was not enough to meet the N requirement of soybean. The initial amount of 0.19% N at the study location (Table 1) is classified as low by Landon (2014). Nitrogen requirement of soybean is relatively higher compared to other grain legumes (George & Singleton, 1992) and yield responses will not occur, even when superior rhizobia inoculants are applied, in soils where nitrogen is not limiting (Catroux, Hartmann & Revellin, 2001). The low nitrogen conditions allowed for optimal performance of the introduced rhizobia strains.

Chlorophyll content serves as an index for nitrogen content in the plant. Bojović & Marković (2009) reported a close link between chlorophyll content and nitrogen in wheat. In this study, chlorophyll content significantly explained 50% of the variation in soybean grain yield in 2018, indicating the effectiveness of rhizobia strains. Among the treatments, only inoculated soybean plants recorded high chloropyll contents.

Greater yield response to combined application of inoculant and p-fertilizer indicates that the applied P enhanced soybean response to the rhizobia inoculant. Ulzen et al. (2018), Ronner et al. (2016), Masso et al. (2016), Adjei-Nsiah et al. (2018), Kyei-Boahen et al. (2017), and Ulzen et al. (2020) all reported increases in soybean and cowpea yields to rhizobia inoculation and p-fertilizer. Rhizobia inoculant complement p-fertilizer in legumes. Phosphorus plays important role in nitrogen fixation and the general growth of the plant as it tranfers energy (in the form of adenosine triophosphate (ATP) required for nodulation (O’Hara, 2001)). Although the initial P content of the study location was medium (11 mg kg^−1^), response of soybean to the applied P indicates that the initial content was not enough to meet the P requirement of the plant as depicted by the control grain yield.

The VCR indicated that the application of rhizobium inoculant and/or p-fertilizer was profitable in 2018 and 2020 using the threshold of two indicated by Roy et al. (2006). Farmers in Ghana could realise returns of 4, 5–8, and 4–25 folds of every amount invested in p-fertilizer alone, inoculant and p-fertilizer, and inoculant alone respectively. Even if we used a higher threshold of 3–4 as proposed by Dittoh et al. (2012) as an index of profitability, a farmer would make 100% profit with the application of p-fertilizer alone, 125–200% profit with combined application of inoculant and p-fertilizer and 100–625% with the application of inoculant alone. Although the combined application of inoculant and p-fertilizer had a higher yield than the inoculated treatment alone in 2018, the value cost of the inoculated treatment was more than thrice that of the combined application of the inoculant and p-fertilizer. This is explained by the additional cost in the combined treatment as VCR is indirectly proportional to the cost of inputs. Smallholder farmers are risk-averse and are likely not to invest in co-application of inoculant and p-fertilizer due to the larger cost associated with it, even though, the benefit is potentially larger.

The inability of rhizobia of the control plots to form nodules was expected as it was the first-time soybean was planted in the area, although indigenous rhizobia existed in the study location (Date, 2000). This implies that nutrition in terms of energy for symbiosis was low in the control plots as p-treated plots formed nodules. The rhizobia count and grain yield in 2019 suggests that the introduced strains persisted. Even though, rhizobia persistance reduces the frequency and cost of inoculation the lack of response and/or the low yields recorded in 2019 suggests that reinoculation after the first cropping season is beneficial.

Even though the residual effect of the treatment on maize was not significant, the yields produced were comparable to yields obtained under mineral and organic fertilizer trials (Essel et al., 2020) and about double the current national average yield of 2.26 t ha^−1^ (Ministry of Food & Agriculture, 2018). This indicates that the yield cannot be attributed to the treatment effects alone. Rotational effect and greater N supply explain yield of maize after soybean cultivation (Carsky et al., 1997; Gentry et al., 2001; Sanginga et al., 2001). The net revenue generated from maize yields indicates that farmers can potentially increase their income with minimum input by growing maize after soybean. This rotation results in minimal disturbance of the ecology and environment, since less amount of N is applied.

The residual effect of the treatments was not significant on soybean yields the next season; however, nodules were detected in plant that had been inoculated previously. This could reflect spread of rhizobia from inoculated plots to uninoculated plot. Also, senescence nodules, roots, and fallen leaves, as well as the haulms that were returned to the soil, might have increased the N content of the soil. This will reduce the dependency of host on rhizobia for nitrogen fixation in the second year.

Even though, 2018 showed higher grain and stover yields than 2020, the interaction between treatment and year was not significant. This suggests that response to treatments does not vary with year and is reproducible. In particular, combined application of inoculant and P-fertilizer significantly produced higher yields in both years. Lower rainfall may explain the lower yield in 2020 (Fig. S3). Cumulative rainfall received in 2020 minor season was lower than that of 2018 (Fig. S3). In addition, rainfall seldomly exceeded 10 mm during the flowering stage in the 2020 minor season. This was followed by a 10-day dry period, high rainfall of 43 mm, and a 5-day short dry period again (Fig. S3). Flowering and podding determines soybean yield and dry spells negatively affect soybean yield (Ulzen et al., 2016). The 2020 grain yield results indicate that soybean varieties that can withstand and adapt to drought are needed to combat food insecurity.

## Conclusion

Soybean responded to *Bradyrhizobium* inoculation and/or p-fertilizer on forest soil indicating the potential of the crop to be cultivated in the semi-deciduous forest agro-ecological zone of Ghana. This study has confirmed the significance of *Bradyrhizobium* inoculation and/or p-fertilizer on soybean grain yield. Combined application of inoculant and/p-fertilizer increased yield over the control by 3-fold; this corresponds to 96% of the potential yield of the Jenguma variety. The value cost ratio, and revenues generated from the residual effect of the treatment indicates the potential of increasing farmers’ income in the study location.

## Supplemental Information

10.7717/peerj.12671/supp-1 Supplemental Information 1 Rainfall distribution pattern during the 2018 minor cropping season.

10.7717/peerj.12671/supp-2 Supplemental Information 2 Rainfall distribution pattern during the 2019 major and minor cropping season.

10.7717/peerj.12671/supp-3 Supplemental Information 3 Rainfall distribution pattern during the 2020 minor cropping season.

10.7717/peerj.12671/supp-4 Supplemental Information 4 Raw data: 2018, 2019, 2020 and combined 2018 and 2020 yield and yield parameters and the data on MPN and rainfall data. I+P- Inoculant only I+P+ Inoculant plus P-fertilizer I-P- Control I-P+ P-fertilizer only

## Additional Information and Declarations

### Data Availability

The following information was supplied regarding data availability: The raw measurements are available in the Supplemental File.
