# Supplementary figures and images for: Soybean (Glycine max L. Merrill) responds to phosphorus application and rhizobium inoculation on Acrisols of the semi-deciduous forest agro-ecological zone of Ghana
Source: PeerJ. 2022 Mar 2;10:e12671. doi: 10.7717/peerj.12671 (PMC8898006; doi:10.7717/peerj.12671)

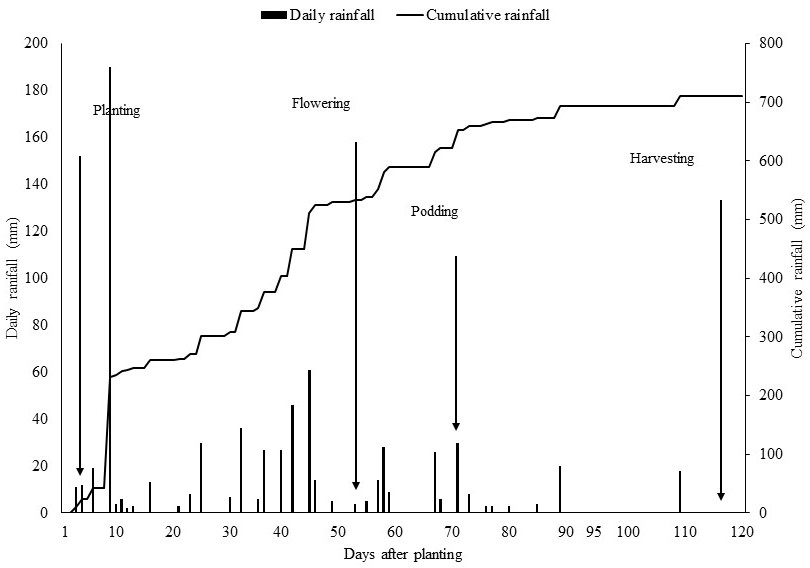

Supplement: Supplemental Information 1 [file peerj-10-12671-s001.jpg]

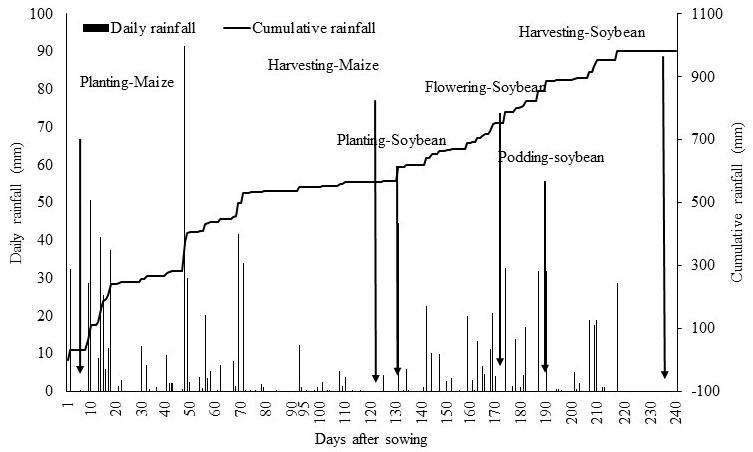

Supplement: Supplemental Information 2 [file peerj-10-12671-s002.jpg]

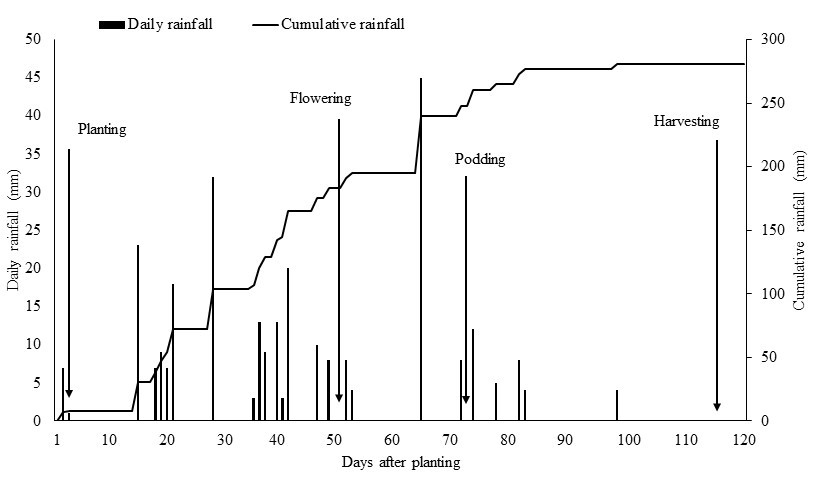

Supplement: Supplemental Information 3 [file peerj-10-12671-s003.jpg]
